# Retrospective Study of Biochemical and Haematological Changes in Diabetes Mellitus

**DOI:** 10.3390/ijms27146179

**Published:** 2026-07-10

**Authors:** Jovita I. Mbah, Phillip T. Bwititi, Lin K. Ong, Prajwal Gyawali, Ezekiel U. Nwose

**Affiliations:** 1 School of Health & Medical Sciences, Centre for Health Research, University of Southern Queensland, Toowoomba, QLD 4350, Australia; lin.ong@unisq.edu.au (L.K.O.); prajwal.gyawali@unisq.edu.au (P.G.); 2 School of Dentistry & Medical Sciences, Charles Sturt University, Wagga Wagga, NSW 2650, Australia; pbwititi@csu.edu.au

**Keywords:** blood flow, diabetes mellitus, FBC HbA1c, estimated whole blood viscosity

## Abstract

Routinely tested biochemical and haematological molecules in the management of diabetes control, including glycated haemoglobin (HbA1c) evaluation, are related to estimated whole blood viscosity (eWBV), which is an indicator of diabetic rheology pathology. Examination of the molecules control could establish their value for clinical laboratory-based cardiovascular risk assessment. The study investigated changes in full blood count (FBC) parameters and lipid profiles with eWBV and HbA1c. The predictiveness of FBC parameters for eWBV and HbA1c is explored. This was a laboratory-based observational study that used mixed-methods analyses. Cross-sectional, longitudinal, descriptive, correlation, and regression approaches were applied to archived clinical pathology data from two health facilities, including a dataset of N = 21,026. Results: Lipid profile molecules significantly change with the level of HbA1c (*p* < 0.001). The levels of eWBV and its determinant molecules also change with HbA1c, and with seasonal variations (*p* < 0.001). Considering the predictiveness of FBC parameters for HbA1c and eWBV levels, red blood cells (RBC) and their indices, i.e., haemoglobin molecules, show statistical significance. For instance, RBC most strongly predicted HbA1c (r = 0.30) and was highest for eWBV (r = 0.54), while mean cell volume (MCV) showed strongest inverse correlation with HbA1c (r = −0.47). Conclusion: This study demonstrates that routine FBC parameters, RBC and its indices, offer a practical, low-cost approach to both monitoring and prediction of HbA1c with blood flow issues. This broadens the understanding of blood rheology in diabetes. Future research should move beyond de-identified laboratory datasets and involve patients’ primary data collection to include medication and clinical information that are unavailable in pathology records.

## 1. Introduction

### Background Information—Diabetes and Complications

Diabetes mellitus affects about 10% of adults globally and continues to be a major public health challenge that is associated with a cluster of metabolic abnormalities, which are preventable risk factors. The disease is characterised by raised levels of blood glucose due to an absence or inadequate insulin levels, or insulin resistance in tissues. The resultant hyperglycemia, if unmanaged, leads to long-term damage and dysfunction of various organs such as the kidneys, heart and blood vessels [1]. Dyslipidaemia is common in diabetes due to insulin resistance or deficiency, affecting major enzymes and pathways in lipid metabolism [2]. There is an imbalance in haematological parameters, one that can be considered abnormal [3], which is driven by hyperglycaemia and dyslipidaemia [3,4]. These abnormalities lead to the development of other diseases; hence, they are a focus for preventive health. For instance, the need to associate red blood cell indices with diabetes has remained imperative [5]. However, this impairment has not yet been considered as part of diabetes management guidelines. This necessitates further evaluation of the association of changes in haematological parameters and haemorrheology (i.e., whole blood viscosity (WBV)) with dyslipidemia and HbA1c.

A narrative established that diabetes mellitus is a metabolic disorder characterised by cellular and molecular disturbances, which manifest as alterations in biochemical, haematological and lipid parameters. Changes in haematology and lipid profile have been reported; however, the pattern of these changes and their correlation has not been elucidated. Dyslipidemia is critical to diabetes complications and needs to be managed to reduce the incidence of CVDs (cardiovascular diseases). Although dietary intervention and exercise can in certain cases reduce diabetic dyslipidemia, many cases will require pharmacological intervention [6]. Correlation between haematological and lipid profiles over the course of diabetes progression, using HbA1c as an index of glucose control, is necessary for additional empirical data and update.

Changes in haematological parameters due to diabetes are reflected in changes in WBV and are related to the development of diabetic complications [7]. For instance, increased differential cell counts, including neutrophils, eosinophils and monocytes, are indicators of the development of coronary artery disease, and WBC (white blood cell) are suggested to play a role in the development and progression of diabetic complications [8]. In a bibliographic review that examined research conducted on WBV in the context of diabetes over the past 20 years with the aim of establishing a framework for current priorities and guiding future research direction [9], the association of abnormal erythrocytes, as well as the relationship between estimated whole blood viscosity (eWBV) and metabolic syndrome, is established. However, there is a dearth of longitudinal study data around WBV in diabetes management. This lack of data calls for further studies, especially prospective and retrospective analyses, to investigate diabetes mellitus with regard to routinely tested biochemical and FBC molecules, as well as eWBV between periods and cohorts.

It is pertinent to acknowledge the complications that arise from complex pathophysiology as well as the individual differences [10]. For instance, underlying complications such as retinopathy, neuropathy, and nephropathy are associated with the production of advanced glycosylated end products. However, assessment of routine FBC and clinical biochemistry parameters is rarely available for review at most laboratory facilities. Yet, it is known that comorbidities such as anaemia and treatment regimen could impact HbA1c level. [11]. Hence, the evaluation of FBC parameters may constitute a significant component of clinical laboratory monitoring.

Objective of study: The aim of this study is to associate routine FBC parameters with diabetes and blood flow, as indicated by eWBV, to generate data that advocates the inclusion of routine haematological parameters in the guidelines for diabetes management. An adjunct to this is to investigate the utility of eWBV and routine haematological molecules in patients with diabetes to optimise management and prevention of complications associated with blood flow.

## 2. Results

### Description of Datasets

The first of two datasets comprised 10 years from 1999 to 2008 ACPD (N = 230,401); after the exclusion of cases with incomplete data, N = 21,026 were included in the study. Table 1 presents a sample of descriptive statistics based on the analysis of specific objective one of the studies.

The dataset described in Table 1 lacked FBC parameters; hence, the second dataset, though much smaller (N = 245), included FBC parameters and was from a general practice in regional NSW, Australia [12]. Table 2 shows the descriptive statistics of the cross-sectional data for routine FBC, eWBV, and HbA1c.

Both datasets constitute two of three sets available in the repository [13]. In this study, the larger first dataset was used to analyse the first and second research objectives, while the smaller dataset with FBC was analysed for the third specific objective.

Comparative analysis: Apart from age and triglyceride being positively related with HbA1c, the levels of cholesterol, HCT and total protein appeared to be inversely associated with changes in HbA1c. The major finding of interest here is that HCT and total protein were statistically significantly decreasing (*p* < 0.0001), especially considering that these two variables are used in extrapolating eWBV, which is expected to increase with HbA1c (Table 3).

Further analysis shows that HCT initially increased with increasing age before declining (Table 4). Average levels of all three variables (haematocrit, protein levels, and viscosity) were significantly different between the stratified age groups (ANOVA, *p* < 0.001). Further, correlation analysis affirmed that blood viscosity measured by eWBV may be inversely associated with HbA1c; gender is a statistically significant (*p* < 0.001) confounding factor. On comparisons of seasonal changes in HbA1c, HCT, serum protein and eWBV levels, all variables are higher during colder seasons, peaking in the winter. However, HbA1c did not achieve statistical significance (Table 5).

Cohort and regression analysis: In a periodic cohort analysis, only RDW shows a significant difference (*p* < 0.04) between cohort groups; however, it is the least correlated with HbA1c changes. On analysis of the change scores, RBC has the strongest positive linearity with HbA1c (r = 0.30), and is among the top three for eWBV (r = 0.54), while MCV has the strongest inverse linearity with HbA1c (r = −0.47). The regression analysis shows that RBC and its indices are predictive for both HbA1c and eWBV, but different variables may be relevant for each case (Table 6).

## 3. Discussions

This study examined the relationships between routinely tested biochemical molecules for glycaemic control (HbA1c), haematological molecules, especially those related to haemoglobin, and indices of eWBV with particular attention to their roles in diabetes. The findings provide new insight into how routinely measured molecules, cells and FBC indices in clinical pathology may contribute to risk stratification and clinical decision-making. The sections are organised into narrative accounts of glycaemic control and haematological–biochemical changes. This is followed by the confounding influence of age and gender on viscosity-related molecules, and afterwards the seasonal variation in the variables. Finally, the predictive value of FBC parameters with special focus on haemoglobin molecules and related RBC indices. Implications for clinical practice are also discussed.

### 3.1. Assessment of Changes in Levels of Molecules with Glycaemic Control

The relationship between glycaemia and haematocrit, serum total protein, and lipid levels possibly plays a role in the pathological process and hence is a subject of interest. The viscosity of blood is associated with various diseases, and rheological parameters are often disturbed in diabetes mellitus. Therefore, detection of microcirculatory disturbances and reversal of rheological impairments are major components of diagnostic and therapeutic strategies in the management of diabetes mellitus [15]. Blood viscosity impairs blood flow and is a factor in other vascular diseases. Glycated haemoglobin is reported to be an independent contributor of blood viscosity and haematocrit, and patients with prediabetes are also reported to have increased blood viscosity and haematocrit [16]. Blood viscosity is correlated with glycated haemoglobin [17], but the mechanism of this association has not been extensively investigated.

In comparison, with worsening glycaemic control, serum levels of high-density lipoprotein cholesterol, total cholesterol, haematocrit, and proteinaemia gradually decrease, while serum triglyceride and age increased (Table 3). In correlation analysis, serum triglyceride level was positively correlated with glycated haemoglobin, while haematocrit and proteinaemia are negatively related. HbA1c may influence whole blood viscosity through mechanisms beyond haematocrit. Its inverse association with haematocrit and proteinaemia suggests additional modifiers such as age, triglycerides, or other haematological factors. These relationships indicate that viscosity does not always rise in parallel with poor glycaemic control.

Notably, the observed inverse relationship between HbA1c and both haematocrit and serum protein suggests that changes in blood viscosity are not driven solely by standard determinants such as cell volume or plasma protein concentration. Instead, glycaemic status may influence rheological properties through additional mechanisms, including erythrocyte deformability, aggregation, and oxidative stress. This highlights the complexity of interpreting eWBV in diabetes and suggests that its relationship with HbA1c is not strictly linear.

### 3.2. Confounding Effects of Age and Gender in eWBV Determinant Molecules

Blood viscosity is central to blood flow and vascular resistance; it increases in diabetes and is inversely related to flow. Haematocrit and serum total protein—core pathology markers of blood stasis—decline with age, yet inexpensive laboratory indicators of stasis remain underused in clinical practice. Hemorheological abnormalities are linked to age-related chronic diseases, including metabolic syndrome, cardiovascular disease, and diabetes mellitus [16,18]. Haematocrit and serum total protein, major determinants of whole blood viscosity (with serum proteins and HCT as surrogates for haemoglobin molecules), show clear age-related variation. Haematocrit predicts poor prognosis in acute ischaemic stroke and transient ischaemic attack [19], and its age-related decline contributes to the high prevalence of anaemia in older adults [20]. Gender differences are also evident, with women exhibiting lower haematocrit and viscosity than men [21].

Average proteinaemia, haematocrit, and eWBV varied across age groups and were consistently lower in females. MANOVA confirmed significant differences between younger adults and the elderly. Although age correlated inversely with eWBV, the association was weak in diabetes. Haematocrit and eWBV initially rose before declining, raising questions about the age at which this downward shift begins. The inverse association between age and markers of blood pooling aligns with established links between ageing, anaemia, hypo-proteinaemia, and hypo-viscosity.

These findings demonstrate clear age- and gender-related differences in the pathology underlying blood viscosity. Lower viscosity in women and older adults implies a comparatively higher bleeding risk. Lower viscosity complicates assumptions about thrombosis risk and influences how antiplatelet prophylaxis should be considered in older adults.

### 3.3. Impact of Seasonal Variations in Blood Viscosity and HbA1c

WBV is the resistance of blood flow through vessels and is influenced by factors such as haematocrit, plasma proteins, red cell properties of deformation and aggregation [22,23]. Elevated level of blood stasis (i.e., accessible by eWBV) is a known contributor to cardiovascular risk [24]; hence, understanding eWBV is crucial for clinical management of cardiovascular risk [25]. Moreover, CVDs are associated with seasonal variation, and the potential impact of seasonal changes on blood viscosity and associated biomolecules poses substantial cardiovascular risk and is therefore a subject of interest.

Seasonal changes show that whole blood viscosity, haematocrit, and serum total protein levels increased with colder seasons, with a peak in winter. However, the seasonal variation in the level of glycated haemoglobin did not achieve statistical significance. Seasonal elevation in eWBV, haematocrit and serum total proteins are consistent with studies reporting increased plasma viscosity and hypercoagulability in colder months [26,27]. Blood viscosity fluctuates between seasons, with peak occurring in the winter season. This fluctuation will assist clinicians to adjust monitoring and treatment strategies for diabetic risks seasonally. In addition, recognition of seasonal variations will help in precise risk assessment and timely interventions to mitigate the risk of cardiovascular events in diabetes management.

The seasonal fluctuation in viscosity-related parameters suggests that environmental factors influence haemorrheological status independently of glycaemic control. This has important clinical implications, as it indicates that cardiovascular risk assessment in diabetes may benefit from seasonal context. Incorporating seasonal awareness into monitoring strategies could improve the timing and precision of preventive interventions.

### 3.4. Regression Analysis for Predictiveness of Haematological Parameters

Routine FBC is part of the haematological workup in clinical diabetes management, even though it is not included in the guideline recommendation for diabetic management. [28]. While associations and predictions are different concepts, given the clarifications in recent times [29,30], with the former being mostly investigated in research, there is a dearth of information regarding predictive values of each haematological parameter for diabetes control (HbA1c) and its blood viscosity complications.

In this investigation of how each parameter of routine FBC may be associated and/or predictive of HbA1c and estimated whole blood viscosity (eWBV), it is important to consider that HbA1c and diabetes are related by haemoglobin (Hb) glycation and clinical management of diabetes, with the prophylactic antiplatelet being part of preventive CVD medicine and underpinning the thrombosis and haemostasis criteria for major bleeding [31].

It was observed that HbA1c, RDW, MCV, and RPR were statistically significantly different between HbA1c groups. All three parameters, as well as MCH and RBC, are significant in the regression analysis. On the eWBV, 5/7 parameters (HCT, HB, RBC, WBC, and MLR) are significantly associated in regression. This study affirms haematological parameters that could be either associative and/or predictive for HbA1c and eWBV [29]. The implications for review of prognostic values of FBC variables in diabetes management are discussed.

### 3.5. Correlation of Whole Blood Viscosity with Blood Cell Indices

Red blood cell (RBC) indices, but neither platelet nor lymphocyte ratios, have been part of routine haematology tests in clinical medicine, including diabetes management. The risk of bleeding is part of blood flow pathophysiology in diabetes mellitus (DM), and there may be a potential for the relationship between blood cell indices and estimated whole blood viscosity (eWBV) in DM. Whole blood viscosity (WBV) is the inherent resistance of blood to flow and is influenced by factors such as red blood cell (RBC) count (haematocrit), red cell properties of aggregation and deformability, and blood protein levels [32].

Changes in blood viscosity in diabetes are related to structural changes in haemoglobin molecules, osmotic disturbances and cytoplasmic viscosity within the cells [33]. Most parameters falling within normal ranges suggest that the study population did not exhibit overt haematologic abnormalities at baseline. The two exceptions are elevated HbA1c, which is a reflection of poor control and increased risk of complications, while marginally high MLR may indicate subtle metabolic and inflammatory activity/early stage of cardiovascular event [34]. The periodic-cohort comparison identified RDW as the only parameter showing a statistically significant difference between groups. This could possibly be an indication of the presence of systemic inflammation [35]. The positive correlation between RBC count and both HbA1c and estimated whole blood viscosity (eWBV) suggests that rising glycaemia may be accompanied by increased blood viscosity, which is known to worsen microvascular perfusion and may contribute to diabetic complications.

The ongoing clinical use of RBC variables is superior compared to profiles of platelet and/or lymphocyte ratios in assessing potential risk of bleeding (i.e., hypo-viscosity) in diabetes. The interesting observation around red cell distribution requires further investigation. Knowledge of the ratios has been of research interest in areas of intracranial bleeding [36,37].

### 3.6. Significance of Study

Diabetes mellitus diagnosis: HbA1c is directly linked to diabetes mellitus as it measures the extent of Hb glycation. This glycation of Hb highlights the importance of haematology in diabetes diagnosis. Blood cell changes and estimated whole blood viscosity offer early, low-cost clues to diabetes. Altered red cell deformability, raised HbA1c, and elevated white cell counts reflect impaired glucose control and inflammation. Increased blood viscosity signals microvascular stress, helping identify undiagnosed diabetes and individuals at higher risk of complications. This study demonstrates that haematological parameters provide a cost-effective, accessible alternative for monitoring diabetic complications, particularly in low-resource settings [38]. It advances understanding of the eWBV method and clarifies the complex relationship between HbA1c, HCT, and WBV [16], supporting more accurate cardiovascular risk assessment. The findings strengthen clinical decision-making, enhance early detection efforts, and inform health promotion initiatives and future research in diabetes management. It will also raise awareness of the utility of haematological analysis in patients with diabetes to optimise management and prevention of associated complications.

Monitoring of diabetes mellitus progression: It has been suggested that an abnormal HbA1c warrants further investigation of the full blood count (FBC), particularly the red blood cell indices [39]. What this report contributes is an updated perspective that reinforces the need for individualised reference interpretation in medical practice. The relevance of personalised care in diabetes management is well established [40] and this paper extends that principle into the domain of clinical laboratory medicine. Specifically, the observed variability in correlations and statistical significance across FBC parameters suggests that case-by-case interpretation of patient results may offer greater clinical value than reliance on population-based reference intervals. This report offers new insight into their potential prognostic value and emphasises eWBV as a practical marker of stasis.

Monitoring of treatment of CVD in diabetes management: This study enhances understanding of eWBV by demonstrating that eWBV, haematocrit, and serum total protein levels exhibit significant seasonal variation, with higher levels in winter. These findings challenge assumptions that HbA1c and haematocrit consistently increase viscosity [41,42], showing instead that HbA1c may be inversely related to haematocrit, complicating interpretation. The observed winter rise in viscosity-related markers aligns with increased cardiovascular risk during colder months, particularly in individuals with diabetes [27]. By highlighting the influence of seasonal physiology, the study underscores the need for season-specific reference values and more nuanced cardiovascular risk assessment in clinical practice.

Monitoring CVD in diabetes management: Effective monitoring of chronic conditions such as CVD and peripheral artery disease involves the assessment of risk factors. It has been suggested that HbA1c may be useful in identifying individuals at risk of blood flow pathology [16]. This study highlights the clinical importance of seasonal variation in haematological parameters, demonstrating that eWBV, haematocrit, and serum total protein levels peak during winter, when cardiovascular morbidity is highest. Indeed, the concept of high WBV being associated with diabetes has been of interest [22]. By establishing eWBV as a practical, cost-effective indicator of blood viscosity, the study expands opportunities for cardiovascular risk assessment in diabetes, particularly in low-resource settings. It reinforces the value of individualised interpretation of FBC parameters and supports their integration into diabetes monitoring, early complication detection, and self-management programmes. The findings also advance the recognition of eWBV as an accessible marker of stasis within thrombophilia pathophysiology, strengthening personalised clinical care.

Strength and limitations of study:

The strengths and limitations of this study are as previously published [43]. However, it is worth reiterating that this study has generated empirical data that could be used to advocate for the inclusion of haematological parameters in the guidelines for DM diagnosis, as well as monitoring of DM progression, treatment, and CVD management. The strength of this study lies in its applicability to diabetic populations, where routine FBC and total protein tests are accessible.

The relevance of this research topic is due to the sharp increase in the incidence of type 2 diabetes mellitus, metabolic diseases, and their complications worldwide, as well as the urgent need to find new approaches to their effective prognosis and management. This retrospective study contributes to a deeper understanding of blood rheology and has important implications for clinical practice in diabetes.

In terms of limitations, it is important to acknowledge that individual differences apply [10]. We acknowledge and recall the point that underlying complications such as retinopathy, neuropathy, and nephropathy are the production of advanced glycosylated end products (AGEs). However, the scope of this study was limited to the investigation of changes in FBC parameters and lipid profiles in relation to eWBV and HbA1c through a retrospective review of available laboratory data.

For instance, it is known that comorbidities such as anaemia and treatment regimen could impact HbA1c levels [11]. Hence, the evaluation of FBC parameters may not be extrapolated to the whole diabetic population but constitute significant clinical laboratory monitoring.

One of the strengths of the study is the additional clinical potential of routine FBC tests that are already available. That is, this study neither recommends any new test that would cost patients and/or health services, nor does it recommend that clinicians rely solely on these indices in routine clinical practice. Yet, it is recommended that future research should recruit broader representative populations and consider evaluating data that are outside the scope of laboratory information systems.

## 4. Methods

STROBE compliance: The indications of compliance with STROBE guidelines are as published [43] and further outlined hereunder.

Design: This was a laboratory-based observational study, which employed mixed quantitative methods, including cross-sectional, retrospective cohort, and regression analyses of archived clinical data. The Akaike Information Criterion regression modelling design was chosen to identify routine haematological parameters that best predict HbA1c and eWBV.

Setting: All laboratory data were collected from two sites in regional New South Wales (NSW), Australia. The data comprised a larger dataset from public pathology in South West NSW [44] and a smaller dataset from private general practice in Central West NSW [12].

Participants: Deidentified pathology data of diabetes patients were used as previously reported [10,43,45]. Data mining was based on HbA1c test and assumed to be predominantly type 2 DM. This study involved no contact with individual participants.

Data variables: 18 variables included six independent FBC parameters (haematocrit, haemoglobin, red cell distribution width (RDW), platelet count, RBC, and WBC) and two independent clinical biochemistry (serum total protein levels, and HbA1c). The remaining ten dependent variables were as described in previous publications [46].

Selection criteria: Inclusion criteria were data that had a full blood count (FBC), HbA1c, and serum protein results. The exclusion criterion was incompleteness of data, as previously published [10].

Study size: The datasets were two, including a 10-year dataset of N = 21,026 from the public pathology LIS [43], and the second set of N = 244 from private general practice [46].

Statistical analysis: This study included five mixed-methods quantitative analyses [10,43,45,46,47] and are listed in Table 7. Thus, different groups were defined according to the specific objectives of each analysis. In the regression analysis, the AIC model was adopted as it determines the best fit among a plethora of complex variables. Variables that are retained as statistically significant for potential predictive value are based on stringent and robust model selection. Statistical software is clarified to include three models—Excel analysis toolPAK Microsoft 365, IBM SPSS statistics version 29, and ‘R’ version 4.5.2.

Ethical clearance: All data were mined from LIS and had ethical clearance from pathology [44] and from Charles Sturt University’s Human Research Ethics Committee (Ref #: 2014/158).

## 5. Conclusions

This study showed that routine haematological parameters, especially haemoglobin and the associated RBC indices, provide a practical, low-cost way to monitor diabetes and assess the risk of blood flow pathophysiology. By demonstrating clear seasonal variation and highlighting the need for individualised interpretation of FBC results, it strengthens the understanding of how blood rheology changes in diabetes. These insights support more accurate risk assessment and recognition of either bleeding or thrombotic complications. They also emphasise that widely available tests can guide better clinical decision-making, which is particularly valuable in resource-limited settings where access to advanced diagnostics may be restricted. The impact of age in the overall assessment of cardiovascular risk in diabetes should not be overlooked, and the therapeutic regimen should be structured to reflect the individual’s age.

## Figures and Tables

**Table 1:** Descriptive statistics of dataset 1.

Variable	N *	Mean	Sd	Skewness	Minimum	Maximum
Age	12,986	61.35	13.78	−0.51	8	97
Total chol	12,985	4.94	1.1	0.74	1.3	16.6
Triglyceride	12,968	1.96	1.61	9.75	0.17	62.09
HDL	12,986	1.22	0.38	1.05	0.08	3.7
HbA1c	12,986	6.78	1.61	1.52	2.4	18.6
Sr Protein	12,982	72.11	5.2	0.04	44	109
HCT	12,986	0.43	0.04	−0.43	0.18	0.58

* Among these data, the valid N/23,0401 complete with all variables is N = 12,963.

**Table 2:** Descriptive statistics of dataset 2.

	Mean	Median	Mode	SD	Skewness	Minimum	Maximum
HbA1c (%)	7.67	7.10	5.70	1.87	1.16	4.80	13.60
eWBV (mPas)	17.18	17.22	17.05	0.90	−0.09	13.79	19.66
Hgb (g/L)	148.01	148.00	152.00	15.73	−0.28	82.00	196.00
RBC (10^6^/µL)	5.02	5.00	5.30	0.48	−0.24	3.50	6.20
HCT	0.45	0.44	0.43	0.04	−0.26	0.30	0.55
MCV (fL)	88.91	90.00	91.00	4.14	−0.41	71.00	103.00
MCH (pg)	29.55	29.70	29.70	1.79	−1.25	19.60	32.90
MCHC (g/dL)	332.55	333.00	331.00	10.97	−0.66	277.00	360.00
RDW (%)	13.21	13.00	12.80	1.19	2.27	11.10	20.30
WBC 10^3^*/*µL	7.85	7.50	7.40	2.16	0.68	4.00	14.10
Platelets 10^3^*/*µL	264.66	251.00	246.00	66.10	0.79	138.00	475.00

**Table 3:** Levels of variables compared between HBA1c groups *.

Theme	Variables	Group	Mean	Significance
Confounder	Age (years)	Good	55.1778	*p* < 0.0001 ^‡^
		Moderate	62.7448	
		Poor	63.1182	
eWBV indices	Haematocrit (decimal)	Good	0.4321	*p* < 0.0001 ^†^
		Moderate	0.4318	
		Poor	0.4267	
	Serum Protein (g/L)	Good	72.8615	*p* < 0.0001
		Moderate	72.3801	
		Poor	71.6015	
Lipid profile	HDL-Cholesterol mmol/L	Good	1.2960	*p* < 0.0001
		Moderate	1.2581	
		Poor	1.1566	
	Total Cholesterol mmol/L	Good	5.0765	*p* < 0.02
		Moderate	5.0113	
		Poor	4.8395	
	Triglyceride mmol/L	Good	1.6959	*p* < 0.0001
		Moderate	1.8378	
		Poor	2.1506	

* HbA1c groupings were based on: good control, ≤5.6%; moderate control, 5.7–6.4%; and poor control, ≥6.5%. ^‡^ No statistically significant difference between moderate vs. poor HbA1c groups. ^†^ No statistically significant difference between good vs. moderate HbA1c groups.

**Table 4:** Descriptive statistics of data variables.

	Group	N	Mean	SD
Serum protein levels (*p* < 0.001)	≤29 years	859	73.02	6.39
	30–39 years	1070	72.77	5.66
	40–49 years	2396	72.24	5.30
	50–59 years	4281	72.18	5.46
	60–69 years	5543	71.46	5.47
	70–79 years	4696	70.96	5.83
	≥80 years	2176	69.98	6.43
Haematocrit levels (*p* < 0.001)	≤29 years	859	0.423	0.044
	30–39 years	1070	0.427	0.041
	40–49 years	2396	0.431	0.039
	50–59 years	4284	0.432	0.041
	60–69 years	5544	0.428	0.043
	70–79 years	4697	0.417	0.047
	≥80 years	2176	0.400	0.050
eWBV * (*p* < 0.001)	≤29 years	859	17.13	1.39
	30–39 years	1070	17.14	1.20
	40–49 years	2396	17.10	1.10
	50–59 years	4281	17.10	1.12
	60–69 years	5543	16.93	1.14
	70–79 years	4696	16.72	1.27
	≥80 years	2176	16.35	1.39

Keys: N—sample size, SD—standard deviation, * eWBV extrapolated according to algorithm [14].

**Table 5:** Impact of seasonal variations as confounding factor.

Variables	Seasons (Groups)	Mean	Std. Deviation	N	*p* Values
HbA1c	Spring	6.87	1.753	4668	0.2
	Summer	6.821	1.716	5373	
	Autumn	6.84	1.762	5163	
	Winter	6.878	1.826	5812	
Serum Protein	Spring	71.425	5.957	4667	0.001
	Summer	71.123	5.637	5372	
	Autumn	71.469	5.596	5162	
	Winter	72.149	5.73	5810	
Haematocrit	Spring	0.422	0.045	4668	0.001
	Summer	0.421	0.044	5373	
	Autumn	0.425	0.045	5163	
	Winter	0.425	0.045	5812	
eWBV	Spring	16.854	1.266	4667	0.001
	Summer	16.787	1.189	5372	
	Autumn	16.903	1.215	5162	
	Winter	17.015	1.232	5810	

**Table 6:** Akaike (AIC) regression for predictiveness of FBC parameters for HbA1c and eWBV.

	Predictiveness of Glycaemic Control			Predictiveness of Blood Flow Pathophysiology		
Parameter	AIC for HbA1c	*p* Values	Inference	AIC for eWBV	*p* Value	Inference
HCT	825.89	0.83		426.02	0.00	Erythoapheresis
Hgb	825.91	0.87		431.62	0.00	
MCH	821.59	0.04	When anaemia confounds HbA1c	494.50	0.03	
MCHC	825.36	0.45		498.68	0.38	
MCV	816.53	0.00	When anaemia confounds HbA1c	499.26	0.68	
MLR	825.88	0.80		496.14	0.07	
NLR	824.67	0.26		497.60	0.18	
Platelet	824.04	0.17		498.57	0.35	
PLR	824.48	0.23		499.42	0.94	
PRR	825.92	0.90		496.68	0.10	
PWR	825.75	0.66		498.70	0.40	
RBC	823.31	0.10	When anaemia confounds HbA1c	433.42	0.00	Erythoapheresis
RDW	821.91	0.05		498.96	0.50	
RPR	822.09	0.05		497.68	0.19	
WBC	825.94	0.98		495.31	0.04	Leucapheresis

**Table 7:** Summary of hypotheses and the statistical mixed-methods designs.

SN	Specific Research Objectives	Hypotheses (H0: Null)	Study Design
1	Assess changes and correlations in haematological and lipid profiles in diabetes progression using HbA1c as an index.	No difference in haem and lipid levels between HbA1c groups in cross-sectional data	Cross-sectional, Descriptive & correlation
2	To investigate the epidemiology of eWBV as laboratory evidence-base for CVD in diabetes, in terms of the impacts of age, gender, and comorbidities.	No difference in eWBV levels between age or gender in subgroups of HCT and/or serum protein	Descriptive
		No significant difference in eWBV levels between summer (daylight saving) & winter periods	Longitudinal
3	To evaluate how haematology parameters and lipid profiles confound HbA1c level among individuals with ‘normal versus abnormal’ blood glucose.	Considering instances of discrepancy between HbA1c and BGL results, there is no significant difference in RBC indices between the groups with/without discrepant results	Comparative and regression analyses
		No significant correlation between RBC indices and HbA1 changes	Cohort study and correlation

## Data Availability

The data presented in this study are available in Figshare at https://doi.org/10.6084/m9.figshare.30904331 [13].
